# The Prevalence and Incidence of Atrial Fibrillation in Patients with Acute Pulmonary Embolism

**DOI:** 10.1371/journal.pone.0150448

**Published:** 2016-03-01

**Authors:** Austin Chin Chwan Ng, Dona Adikari, David Yuan, Jerrett K. Lau, Andy Sze Chiang Yong, Vincent Chow, Leonard Kritharides

**Affiliations:** Cardiology Department, The University of Sydney, Concord, New South Wales, Australia; IIBB-CSIC-IDIBAPS, SPAIN

## Abstract

**Background:**

Symptomatic pulmonary embolism (PE) is a major cause of cardiovascular death and morbidity. Estimated prevalence and incidence of atrial fibrillation (AF) in developed countries are between 388–661 per 100,000, and 90–123 per 100,000 person-years respectively. However, the prevalence and incidence of AF in patients presenting with an acute PE and its predictors are not clear.

**Methods:**

Individual patient clinical details were retrieved from a database containing all confirmed acute PE presentations to a tertiary institution from 2001–2012. Prevalence and incidence of AF was tracked from a population registry by systematically searching for AF during any hospital admission (2000–2013) based on International Classification of Disease (ICD-10) code.

**Results:**

Of the 1,142 patients included in this study, 935 (81.9%) had no AF during index PE admission whilst 207 patients had documented baseline AF (prevalence rate 18,126 per 100,000; age-adjusted 4,672 per 100,000). Of the 935 patients without AF, 126 developed AF post-PE (incidence rate 2,778 per 100,000 person-years; age-adjusted 984 per 100,000 person-years). Mean time from PE to subsequent AF was 3.4 ± 2.9 years. Total mortality (mean follow-up 5.0 ± 3.7 years) was 42% (n = 478): 35% (n = 283), 59% (n = 119) and 60% (n = 76) in the no AF, baseline AF and subsequent AF cohorts respectively. Independent predictors for subsequent AF after acute PE include age (hazard ratio [HR] 1.06, 95% confidence interval [CI] 1.04–1.08, *p*<0.001), history of congestive cardiac failure (HR 1.88, 95% CI 1.12–3.16, *p* = 0.02), diabetes (HR 1.72, 95% CI 1.07–2.77, *p* = 0.02), obstructive sleep apnea (HR 4.83, 1.48–15.8, *p* = 0.009) and day-1 serum sodium level during index PE admission (HR 0.94, 95% CI 0.90–0.98, *p* = 0.002).

**Conclusions:**

Patients presenting with acute PE have a markedly increased age-adjusted prevalence and subsequent incidence of AF. Screening for AF may be of importance post-PE.

## Introduction

Symptomatic PE is the third largest cause of cardiovascular death after coronary artery disease and stroke, occurring in about 100 persons per 100,000 annually [1,2]. Our group previously reported that baseline cardiovascular disease (CVD) was an independent predictor of all-cause mortality post-discharge after acute PE and that a history of atrial fibrillation (AF) and/or flutter without other known CVD was a predictor of adverse outcome during long-term follow-up [3].

Nonvalvular AF is the most common cause of cardioembolic stroke [4]. The mechanism of stroke is understood to be thrombus formation in the fibrillating left atrium or atrial appendage, with subsequent embolization. Similarly, thrombus formation in the right atrium has been suggested as a cause for PE in the context of AF [5]. Spontaneous echo-contrast is the presence of smoke-like echoes within the cardiac chambers and is a marker of a hypercoagulable state due to stasis [6]. Yasuoka et al noted right atrial spontaneous echo-contrast in patients with nonvalvular AF and concluded that it may be a predictive factor for PE. Autopsy studies in patients with AF have also raised the possibility that right atrial thrombosis may lead to PE [7,8]. The worldwide prevalence and incidence of AF in developed countries are estimated at 388–661 per 100,000, and 90–123 per 100,000 person-years respectively [9–11]. To date, the prevalence of AF in patients presenting with acute PE, or the subsequent incidence of AF after an acute PE, is unclear.

The aims of our study were: to assess the prevalence of AF in patients with confirmed acute PE; to determine the incidence and predictors of subsequent AF post-acute PE; and, to assess the effect of AF on the outcomes of patients with PE.

## Materials and Methods

### Study population

Patients admitted with acute PE from our institution (Concord Hospital, Sydney, Australia) has been described previously [3]. For the purpose of this study, consecutive patients admitted with a primary diagnosis of acute PE between 1^st^ July 2001 and 31^st^ December 2012 were identified retrospectively from the PE database. Medical records of all identified patients were reviewed for formal confirmation of the acute PE according to published guidelines [3,12], requiring both documented clinical diagnosis and/or treatment of acute PE, together with an imaging study consistent with the diagnosis. Data were extracted directly from medical records of individual patients by authors A.C.C.N, with assistance from V.C. All medical records of patients were reviewed once during data collection. There was no separate data extraction (i.e. double extraction) following this initial data collection. There was no age criteria applied, nor were the PE classified into provoked or unprovoked events in the database. For patients who presented on more than one occasion with acute PE during the study period, only the initial presentation was included. Non-local state (New South Wales [NSW]) residents were excluded to minimize incomplete tracking of long-term outcomes.

Details of patients’ admission, initial hemodynamic profile and symptoms, blood profile, in-hospital outcomes, comorbidities such as history of cardiovascular disease (including ischemic heart disease, prior coronary artery bypass surgery, congestive cardiac failure, valvular heart disease, prosthetic heart valves, atrial fibrillation/flutter, peripheral vascular disease, stroke), cardiac risk factors (hypertension, hyperlipidemia, diabetes, current or ex-smoker), history of malignancy, chronic pulmonary disease (asthma and/or emphysema), obstructive sleep apnea, dementia, Parkinson’s disease, and chronic renal disease coded by diagnosis-related group based on the International Classification of Disease (ICD-10), were retrieved from the PE database. The Charlson Comorbidity Index (CCI) was used to assess patient’s comorbid status [13]. Atrial flutter was classified as part of the AF spectrum for the purpose of the current study.

Prevalence and incidence of AF was tracked for each patient from a population registry by systematically searching for any hospital admission that included either a primary or secondary diagnosis of AF (ICD-10 code I48) from July 2000 to October 2013 using population-linkage analysis.

Ethics approval was granted by Concord Hospital Ethics Committee (CH62/6/2008-009) and the NSW Population and Health Services Research Ethics Committee (2013/09/479). The Committees granted a waiver of the usual requirement for the consent of the individual to the use of their health information. All patients’ data were de-identified and analyzed anonymously.

### Study outcomes

The mortality outcome of the cohort was tracked from a statewide death registry database. The use of a statewide death registry to obtain outcomes is advantageous as the non-captured deaths during our study period is estimated to be only 0.6% based on known migration rates [14]. A censored date of June 30, 2013 was pre-specified for the study. All death certificates were coded independently by at least 2 reviewers (A.C.C.N., J.L. or L.K.) [15]. Reviewers were blinded to patients’ background comorbidities during coding. Disparities were resolved by consensus.

Cardiovascular death was defined as death due to PE, acute myocardial infarction, heart failure, stroke, cardiac arrest and cardiac-related causes (when more than one cardiac cause of death was recorded). Noncardiovascular death included death due to malignancy, sepsis and dementia. Patients with multiple potential causes of death on their death certificates were classified as “undefined” and labelled as noncardiovascular death for the purposes of the study.

### Statistical analysis

The main PE cohort was divided into three groups: Group 1 (control group) included patients without documented AF throughout the study period (1^st^ July 2000 to 30^th^ October 2013); Group 2 (baseline AF group) included patients with any record of AF within one or more years prior to their index PE admission date; Group 3 (subsequent AF group) included patients without any prior history of AF but developed AF following their index PE.

Age-adjusted prevalence and incidence was calculated with a weighted average of the age-specific crude rates, where the weights were the proportions of persons in the corresponding age groups based on the 2001 NSW standard population [16]. All continuous variables are expressed as mean ± standard deviation, unless otherwise stated, and categorical data given in proportions and percentages. Comparison between groups used one-way ANOVA for continuous variables and comparison between two groups for significant results was performed using independent student *t* test with Bonferroni correction, while χ^2^ tests were used for dichotomous variables. Kaplan-Meier survival methods were used to compare unadjusted survival rates, and display the cumulative incidence of subsequent AF. Cox proportional hazards regression analysis was used to compare survival curves adjusted for age, gender, CCI, patient’s admission hemodynamic and blood profiles. To identify predictors for subsequent AF, patients with baseline AF were excluded from the main cohort. Cox proportional hazards regression analysis was used to identify predictors for subsequent AF. Only univariables with *p*<0.10 were included in the multivariable analysis. To avoid significant co-linearity, only univariable predictors with a correlation coefficient ≤0.7 were chosen for the multivariable modelling. The Harrell’s *C* and Somers’ *D* statistic, which examines discrimination performance, was used to assess the multivariable Cox regression model in predicting subsequent AF following acute PE [17,18]. All analyses were performed using SPSS version 22.0 (IBM, USA) or Stata version 10.1 (StataCorp LP, TX, USA). A 2-tailed probability value <0.05 was considered significant.

## Results

### Prevalence and incidence of AF in patients with PE

**Fig 1:**
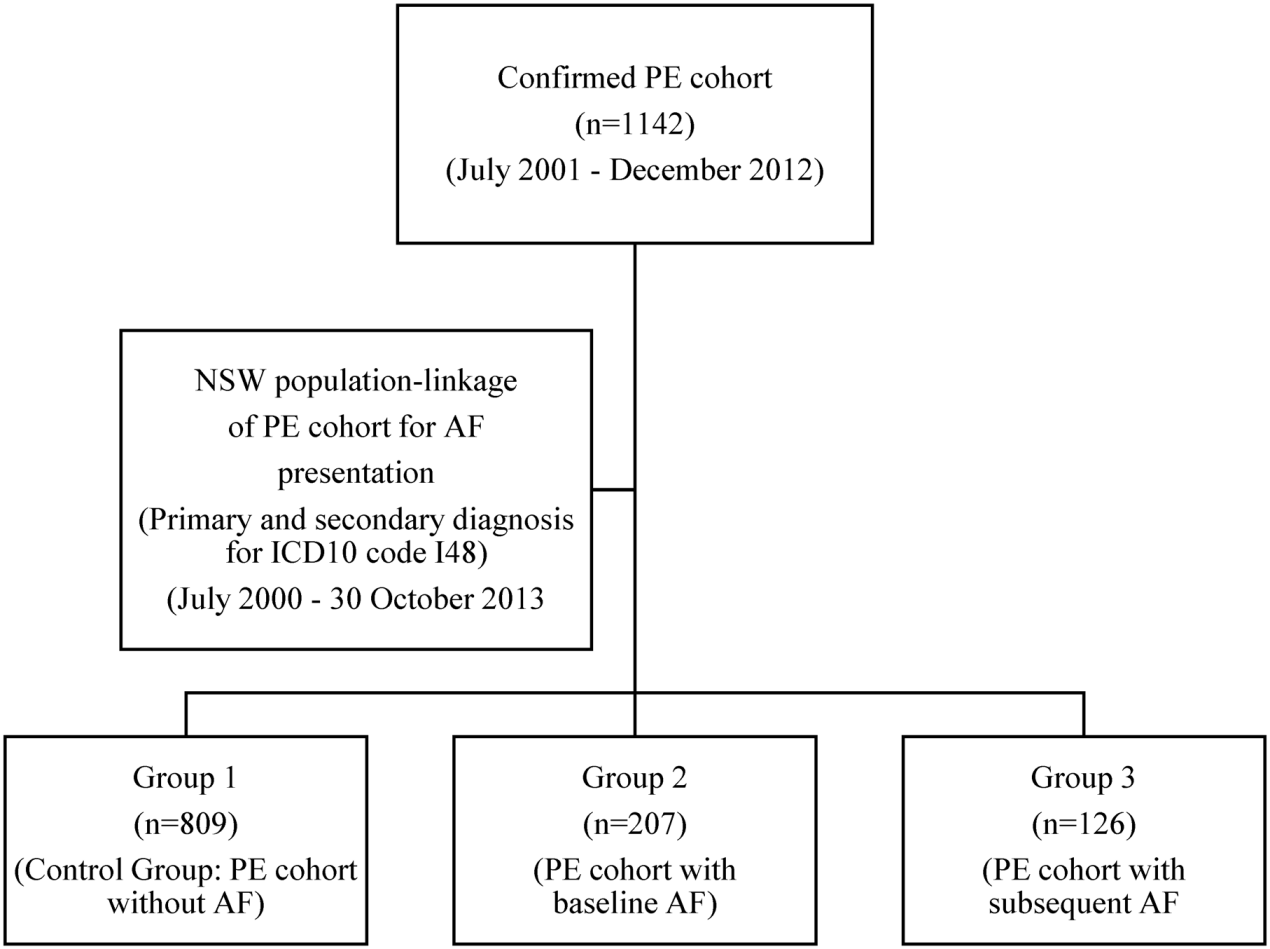
Study flow chart. The flow chart shows the derivation of the three study groups.

**Fig 2:**
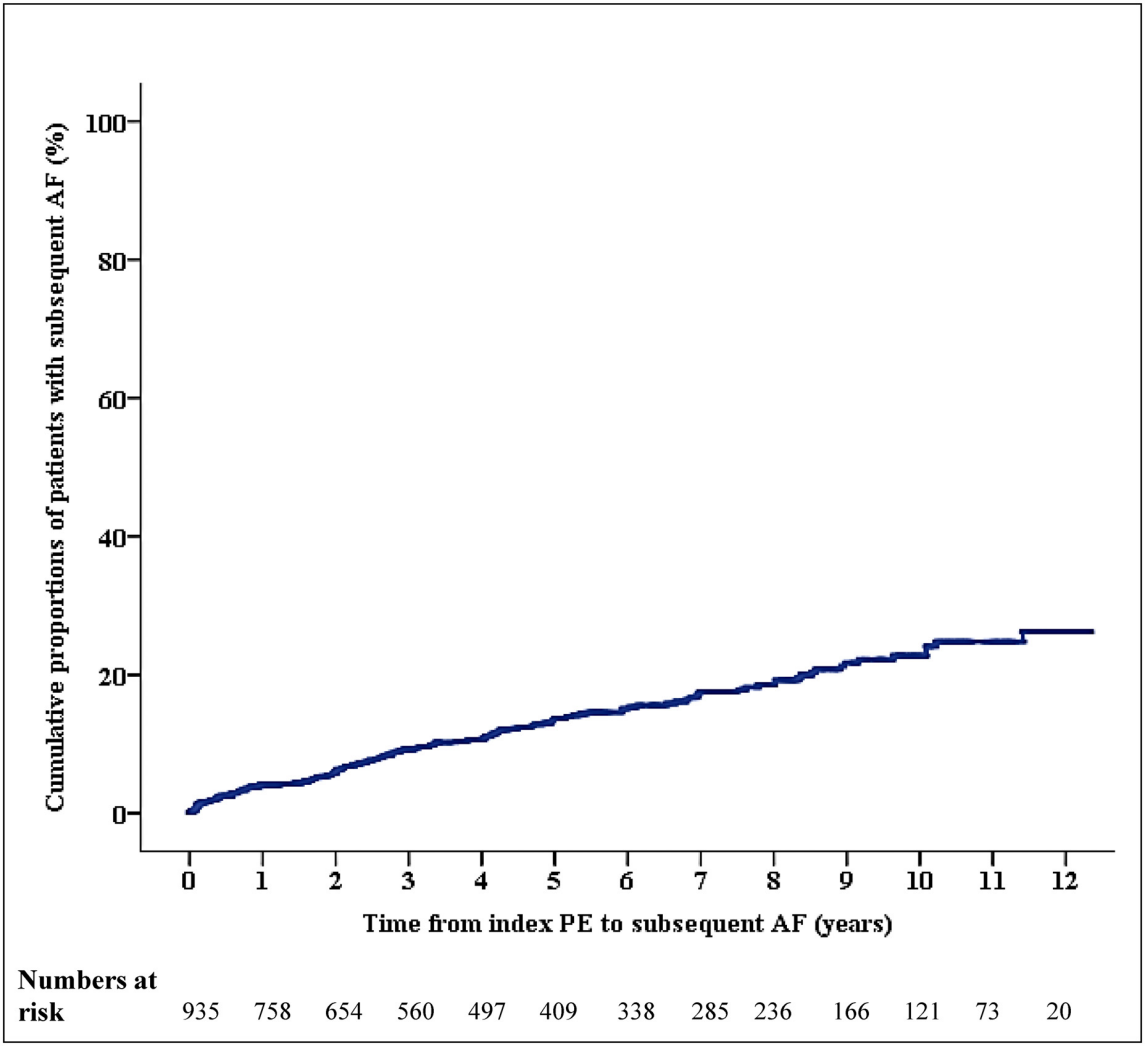
Cumulative incidence of AF following acute PE. The graph shows cumulative proportions of patients in percentages who developed subsequent atrial fibrillation (AF) following the index acute pulmonary embolism (PE). Patients who died during follow up were censored and accounted for in deriving the numbers of at risk patients.

Of the 1,142 patients with confirmed PE included in our study, 207 had baseline AF, while 935 (81.9%) had no documented AF at the time of their index PE admission (Fig 1). The unadjusted prevalence of AF was therefore 181 per 1000 (18,126 per 100,000). The age-adjusted prevalence of AF was 4,672 per 100,000 for the total cohort, and was higher in women than men (5,358 per 100,000 vs 4,218 per 100,000 respectively). Of the 935 patients without baseline AF, 126 subsequently developed AF during a mean follow-up of 5.0 ± 3.7 years (Fig 2). Mean time from PE to subsequent AF was 3.4 ± 2.9 years. The unadjusted incidence of AF post-PE was 2,778 per 100,000 person-years. The age-adjusted incidence rate was 984 per 100,000 person-years for the total cohort, with a slightly higher incidence in men than women (1,167 per 100,000 person-years vs 840 per 100,000 person-years respectively).

### Baseline characteristics across groups

Table 1 shows the baseline characteristics of the study groups. The mean age of the whole cohort was 67.2 ± 16.6 years, with more females than males (55% vs 45% respectively). Patients with baseline or subsequent AF were significantly older than patients without AF (Group 2: 77.5 ± 9.7 vs Group 1: 63.5 ± 17.2 years, *p*<0.05; Group 3: 74.4 ± 11.4 vs Group 1: 63.5 ± 17.2 years, *p*<0.05) and had more comorbidities, reflected in higher mean CCI scores (Group 2 vs Group 1, *p*<0.05). Patients with AF (Groups 2 and 3) had more underlying ischemic heart disease, congestive cardiac failure, hypertension and diabetes than patients with no AF. The prevalence of malignancy did not differ between the groups.

**Table 1:** Baseline characteristics.

Parameters	Group 1 No AF (N = 809)	Group 2 Baseline AF (N = 207)	Group 3 Subsequent AF (N = 126)
Age, years	63.5 ± 17.2	77.5 ± 9.7*	74.4 ± 11.4*
Males, no. (%)	362 (45)	84 (41)	64 (51)
Recorded DVT during admission, no. (%)	184 (23)	34 (16)*	27 (21)
Length of hospital stay, days	7.1 ± 6.2	9.6 ± 8.0*	8.3 ± 5.1
Echocardiogram during admission, no. (%)	308 (38)	110 (53)*	59 (47)
**Imaging modality**			
Ventilation-perfusion scintigraphy, no. (%)	536 (66)	155 (75)*	110 (87)*†
High probability, no. (%)	476 (59)	142 (69)*	86 (68)*
Intermediate probability, no. (%)	47 (6)	9 (4)	18 (14)*†
CT pulmonary angiogram, no. (%)	355 (44)	77 (37)	36 (29)*
Main pulmonary artery, no. (%)	127 (16)	21 (10)*	9 (7)*
Segmental and sub-segmental, no. (%)	306 (37)	65 (31)	27 (21)*†
Both imaging modalities used	85 (11)	24 (12)	19 (15)
**Hemodynamic profile on admission**			
Heart rate, beats/minute	88.2 ± 21.2	89.0 ± 21.2	88.2 ± 21.2
Systolic blood pressure, mmHg	138.9 ± 24.2	140.2 ± 23.0	140.0 ± 25.2
Oxygen saturation, %	95.5 ± 4.4	95.5 ± 3.8	95.2 ± 4.8
**Initial presenting symptoms**			
Presyncope	88 (11)	24 (12)	12 (10)
Syncope	48 (6)	15 (7)	3 (2)
Chest pain	443 (55)	106 (51)	71 (56)
Dyspnea	553 (69)	130 (63)	83 (66)
**Comorbidities, no. (%)**			
Cardiovascular disease	188 (23)	169 (82)*	58 (46)*†
Prior myocardial infarction	105 (13)	39 (19)*	32 (25)*
Prior CABG or PCI	35 (4)	17 (8)*	9 (7)
Congestive cardiac failure	44 (5)	51 (25)*	19 (15)*†
Valvular heart disease	13 (2)	10 (5)*	3 (2)
Peripheral vascular disease	68 (8)	21 (10)	16 (13)
Stroke	19 (2)	11 (5)*	4 (3)
Prosthetic valve	2 (0.2)	7 (3)*	1 (1)
**Cardiac risk factors**			
Hypertension	148 (18)	61 (30)*	46 (37)*
Hyperlipidemia	68 (8)	15 (7)	22 (18)*†
Diabetes	91 (11)	31 (15)	28 (22)*
Current smoker	77 (10)	6 (3)*	13 (10)†
Ex-smoker	136 (17)	40 (19)	27 (21)
**Noncardiovascular disease**			
Malignancy	184 (23)	40 (19)	23 (18)
Chronic pulmonary disease	78 (10)	17 (8)	17 (13)
Obstructive sleep apnea	8 (1)	1 (0.5)	4 (3.2)*
Dementia	28 (4)	16 (8)*	0 (0)*†
Parkinson’s disease	12 (2)	3 (1)	2 (2)
Chronic renal disease	37 (5)	18 (9)*	4 (3)†
Charlson Comorbidity Index score	2.9 ± 3.4	3.9 ± 3.2*	3.5 ± 2.8

Plus-minus values indicate mean ± standard deviation.

AF, atrial fibrillation; CT, computed tomography; CABG, coronary artery bypass grafting; PCI, percutaneous coronary intervention; DVT, deep vein thrombosis; PE, pulmonary embolism.

* *p*<0.05 compared to Group 1 (control group)

^†^ *p*<0.05 compared to Group 2 (baseline AF group).

Table 2 compares the baseline blood profiles and medications of the study groups on admission. Patients without AF had significantly higher sodium and eGFR compared to patients with baseline or subsequent AF. Patients with baseline AF had significantly lower hemoglobin level on admission compared to patients with no AF. They also had a significantly higher INR on discharge compared to patients without AF or those with subsequent AF.

**Table 2:** Baseline blood profiles and medications.

Parameters	Group 1 No AF (N = 809)	Group 2 Baseline AF (N = 207)	Group 3 Subsequent AF (N = 126)
**Blood profile on admission**			
Day-1 sodium, mmol/L	139.0 ± 3.6	138.1 ± 4.5*	138.1 ± 4.8*
Day-1 eGFR, ml/min/1.74m^2^	83.6 ± 33.9	66.3 ± 27.9*	73.9 ± 26.2*
Day-1 hemoglobin, g/L	130.5 ± 20.1	126.5 ± 19.5*	129.3 ± 18.1
INR at time of hospital discharge	2.1 ± 0.8	2.4 ± 0.8*	2.2 ± 0.7†
**Medications use on admission, no. (%)**			
Warfarin	65 (8)	18 (9)	4 (3)
Enoxaparin	29 (4)	5 (2)	2 (2)
NOACs	1 (0.1)	0	0
Aspirin	169 (21)	46 (22)	29 (23)
Clopidogrel	43 (5)	9 (4)	9 (7)
Statins	203 (25)	48 (23)	32 (25)
Beta-blocker	138 (17)	36 (17)	19 (15)

Plus-minus values indicate mean ± standard deviation.

NOACs, non-vitamin K antagonist oral anticoagulants (include dabigatran, rivaroxaban and apixaban); INR, international normalized ratio; Estimated glomerular filtration rate (eGFR) = 186 x ([S_CR_/88.4]^-1.154^) x (age)^-0.203^ x (0.742 if female), where estimated GFR = estimated glomerular filtration rate (ml/min/1.73m2), S_CR_ = serum creatinine concentration (μmol/L), and age is expressed in years.

* *p*<0.05 compared to Group 1 (control group)

^†^ *p*<0.05 compared to Group 2 (baseline AF group).

Following admission, 5 (0.6%) patients in the without AF group and 1 (0.5%) in the baseline AF group received thrombolysis treatment for the PE. No patients in the subsequent AF group received thrombolysis. Amongst the 1,105 patients who survived to hospital discharge, 78.2%, 80.3% and 77.0% of patients were discharged on warfarin in the no AF, baseline AF and subsequent AF groups respectively. The rates of enoxaparin or heparin use were 37.6%, 28.3% and 37.3% respectively across the three groups. Only 5 (0.6%) patients in the no AF group were discharged on a non-vitamin K antagonist oral anticoagulant and none in the baseline and subsequent AF groups.

### Predictors for subsequent AF following acute PE

Table 3 shows multivariable analysis performed to identify independent predictors for subsequent AF following acute PE (for univariable predictors, see S1 Table). Age (hazard ratio [HR] 1.06, 95% confidence interval [CI] 1.04–1.08, *p*<0.001), congestive cardiac failure (HR 1.88, 95% CI 1.12–3.16, *p* = 0.02), diabetes (HR 1.72, 95% CI 1.07–2.77, *p* = 0.02), obstructive sleep apnea (HR 4.83, 1.48–15.8, *p* = 0.009) and day-1 serum sodium (HR 0.94, 95% CI 0.90–0.98, *p* = 0.002) were independent predictors of subsequent AF. The *C* statistics for the multivariable model in predicting the occurrence of subsequent AF was 0.76 (*p*<0.001).

**Table 3:** Multivariable independent predictors for subsequent AF following acute PE presentation.

Multivariable modelling*	Hazard ratio	95% CI	*p* value
Age, per-1-year increase	1.06	1.04–1.08	<0.001
Prior myocardial infarction	1.21	0.74–1.97	0.45
Prior CABG or PCI	0.85	0.39–1.87	0.68
Congestive cardiac failure	1.88	1.12–3.16	0.02
Hypertension	0.84	0.54–1.30	0.43
Hyperlipidemia	1.16	0.68–1.98	0.58
Diabetes	1.72	1.07–2.77	0.02
Chronic pulmonary disease	1.21	0.71–2.07	0.48
Obstructive sleep apnea	4.83	1.48–15.8	0.009
Day-1 sodium, per-1mmol/L increase	0.94	0.90–0.98	0.002
Day-1 eGFR, per-1ml/min/1.74m^2^ increase	1.00	1.00–1.01	0.52
Day-1 sodium, per-1mmol/L increase	1.00	0.99–1.01	0.53

AF, atrial fibrillation; CABG, coronary artery bypass grafting; PCI, percutaneous coronary intervention; PE, pulmonary embolism; Estimated glomerular filtration rate (eGFR) = 186 x ([S_CR_/88.4]^-1.154^) x (age)^-0.203^ x (0.742 if female), where estimated GFR = estimated glomerular filtration rate (ml/min/1.73m2), S_CR_ = serum creatinine concentration (μmol/L), and age is expressed in years.

* Only univariables with *p*<0.10 were included in the multivariable analysis. The *C* statistic in predicting subsequent AF following acute PE for the multivariable model that included the variables age, congestive cardiac failure, diabetes, obstructive sleep apnea and day-1 serum sodium level was 0.76 (95% confidence interval [CI] 0.72–0.80, *p*<0.001). The proportional-hazards assumption was satisfied for each independent variables.

### Mortality outcomes of study groups

Table 4 shows the all-cause unadjusted mortality outcomes of the study groups. There were 28 (3.5%) and 9 (4.3%) in-hospital deaths in the no AF and baseline AF groups respectively. The baseline AF group had the highest mortality rate for the first 5 years of follow-up, with mortality rates of 23.7% at 1-year and 45.4% at 5-year follow-up time-points, compared to the no AF group (16.7% and 29.5% respectively) and the subsequent AF group (9.5% and 34.9% respectively). The subsequent AF group saw a rise in mortality during the long-term follow-up period, exceeding both the no AF group at the 5-year time-point (34.9% vs 29.5% respectively) and the baseline AF group by the end of the study period (60.3% vs 57.5% respectively).

**Table 4:** Unadjusted all-cause mortality.

Mortality, no. (%, 95% CI)	Group 1 No AF (N = 809)	Group 2 Baseline AF (N = 207)	Group 3 Subsequent AF (N = 126)
**Short-term**			
In-hospital	28 (3.5, 2.4–5.0)	9 (4.3, 2.3–8.1)	0 (0)
30-day	39 (4.8, 3.6–6.5)	15 (7.2, 4.5–11.6)	0 (0)
3-month	68 (8.4, 6.7–10.5)	25 (12.1, 8.3–17.2)	8 (6.3, 3.3–12.0)
6-month	98 (12.1, 10.0–14.5)	32 (15.5, 11.2–21.0)	10 (7.9, 4.4–14.0)
**Long-term**			
1-year	135 (16.7, 14.2–19.4)	49 (23.7, 18.4–30.0)	12 (9.5, 5.6–15.9)
3-year	206 (25.5, 22.6–28.6)	75 (36.2, 30.0–43.0)	25 (19.8, 13.8–27.7)
5-year	239 (29.5, 26.5–32.8)	94 (45.4, 28.8–52.2)	44 (34.9, 27.2–43.6)
**Total**	283 (35.0, 31.8–38.3)	119 (57.5, 50.7–64.0)	76 (60.3, 51.6–68.4)

AF, atrial fibrillation; CI, confidence interval.

**Fig 3:**
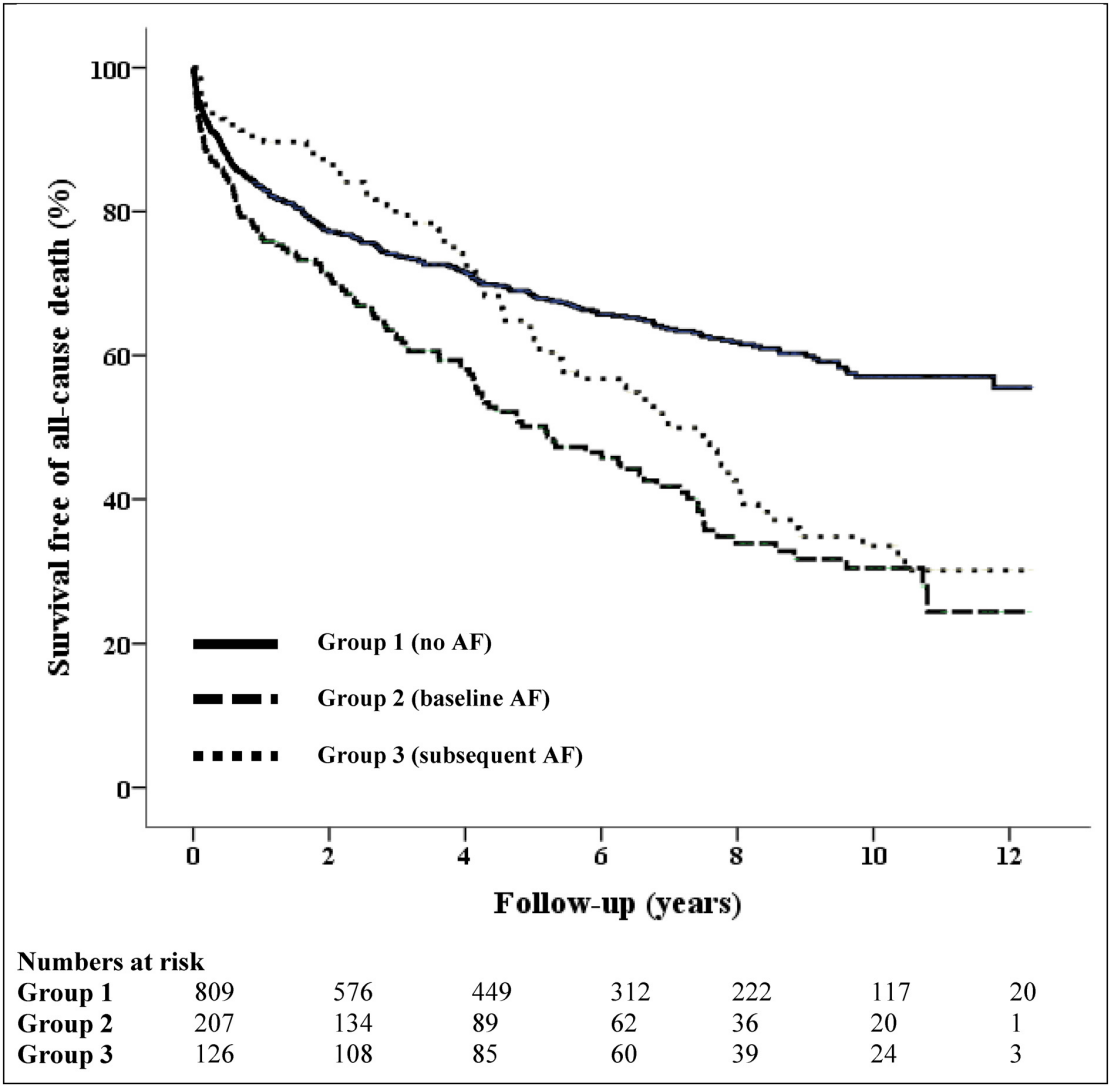
Unadjusted Kaplan-Meier survival curves. The unbroken line (Group 1) shows the survival curve of patients with no atrial fibrillation (AF) at baseline of index PE admission or during study follow-up period. The thick broken line represents patients with known AF at index PE admission (Group 2), and the dotted line represents patients who developed subsequent AF following index PE admission (Group 3). The survival curves differed significantly across all three groups (*p*<0.001).

Fig 3 shows the unadjusted Kaplan-Meier survival curves: patients with baseline or subsequent AF had significantly higher all-cause mortality compared to patients with no AF (HR 1.90, 95% CI 1.54–2.36, *p*<0.001 [Group 2 vs Group 1], and HR 1.51, 95% CI 1.17–1.94, *p* = 0.001 [Group 3 vs Group1] respectively). Using stepwise adjustment initially for age, gender and CCI, baseline AF patients had poorer survival compared to no AF patients (HR 1.29, 95% CI 1.03–1.62, *p* = 0.03), with no difference in survival between subsequent AF and no AF groups. When adjusted for age, gender, comorbidities and patients’ admission hemodynamic profiles, the difference in survival between baseline AF and no AF groups persisted (HR 1.29, 95% CI 1.02–1.63, *p* = 0.03). This difference in survival was still observed between baseline AF and no AF groups though it was no longer statistically significant when additionally adjusted for patients’ admission blood profiles (HR 1.24, 95% CI 0.98–1.58, *p* = 0.08), or with any of the groups (S1 Fig).

### Cause-specific death across groups

Of the total 478 deaths, 35% were attributed to cardiovascular causes, of which PE (10%) was the most common cause, followed by heart failure (8%) and acute myocardial infarction (6%) (S2 Table). Malignancy (29%) and sepsis (22%)) were the two most common noncardiovascular causes. Patients with AF (Groups 2 and 3) had a significantly higher percentage of cardiovascular deaths (50% and 46% respectively) compared to patients with no AF (26%; *p*<0.0001 and *p* = 0.001 respectively). Among patients with baseline AF, PE (13%), heart failure (11%) and acute myocardial infarction (9%) accounted for relatively similar proportions of cardiovascular deaths, while heart failure (17%) was the most common cardiovascular cause in patients with subsequent AF. In contrast, noncardiovascular causes accounted for nearly three-quarters of the deaths in patients with no AF.

## Discussion

The current study shows that in a contemporary population hospitalized with confirmed acute PE there is a substantially increased prevalence of AF and incidence of late onset AF. The age-adjusted prevalence of AF in our PE cohort is 6.4-times higher in men and 13.8-times higher in women than the reported age-adjusted prevalence rates of AF for developed countries in the Global Burden of Disease study by Chugh et al [9]. The present study is the largest to date showing substantially increased incidence of subsequent AF post-PE. Our unadjusted incidence rate of 2,778 per 100,000 person-years is consistent with the only other study on PE patients reported in literature in 2013 by Hald et al with a crude incidence of 2,630 per 100,000 person-years [19]. Our reported age-adjusted incidence rates for subsequent AF in patients who were hospitalized for an acute PE is 9.5-times higher in men and 9.3-times higher in women than the age-adjusted incidence rates reported for developed countries [9].

The unadjusted incidence rate of subsequent AF in the current study is lower than the rate of 7.5% reported by Lopes et al in a pooled analysis of acute coronary syndrome patients [20]. In comparison, our incidence rate appears to be similar to that reported by Psaty et al (2,720–3,930 per 100,000) in their cohort of older adults with CVD [21]. Whilst the incidence of AF in patients with heart failure is not known, the prevalence rate of AF in our PE study is consistent with reported prevalence rates of around 6 to 35% in this high-risk population [22].

An association between PE and subsequent AF has been hypothesized in the literature [5]. On a pathophysiological level, PE may trigger AF by causing acute right ventricular dilatation with strain. It has been suggested that the relation may also be reversed, that thrombus formation in the right atrium can cause PE in patients with AF. Kukla et al reported in a cohort of 1,006 patients with acute PE that AF was detected in 231 (24%) patients and right heart thrombus detected in 50 (5%) patients (16 patients had both AF and right heart thrombus detected) [23]. The fact that deep vein thrombosis was less documented during admission in those patients with baseline AF in our study is suggestive of the role of AF in causing PE in some cases. However, a recent study by Martin et al involving patients with implanted cardiac devices (implanted cardioverter-defibrillators or resynchronization devices) who were monitored for atrial tachyarrhythmia showed no temporal correlation between atrial tachyarrhythmia and clinical thromboembolic events (defined as stroke or systemic embolism) during a median follow-up of 2 years (cohort median CHA_2_DS_2_-VASc score was 4) [24]. It thus seems likely the high prevalence and incidence of AF may be better explained by shared risk factors between PE and AF, including older age, than a direct causal relationship.

This is further supported by the observation that the occurrence of AF following acute PE appears to follow a linear trend in the current study. Mechanistically, there could be two different reasons why patients with PE could develop AF. One is that PE may directly lead to cardiac dysfunctions that, in turn, could trigger AF. The observations made by Hald et al in which the risk of AF was highest in the first six months following a venous thromboembolic (VTE) event would appear to support this [19]. However, a great majority of VTE were deep vein thrombosis (n = 1511) rather than PE (n = 723). The authors did not report if the risk of AF were similarly elevated in the first six months if only PE patients were analyzed. In contrast, our cohort is strictly PE patients and were older. Our observations of a proportionally-linear increased incidence trend for AF following acute PE supports the hypothesis that patients with PE likely share some of the general risk factors for AF development. In PE patients, we found age, a history of congestive heart failure, diabetes and obstructive sleep apnea to be independent predictors for subsequent AF. These variables are established risks factors for AF in the general population [25,26]. Interestingly, we believe we describe for the first time serum sodium during index PE as an independent predictor for AF, such that for every 1-mmol/L higher level of serum sodium on admission, there is a 6% lower risk for subsequent AF. This observed association between low serum sodium and subsequent AF in acute PE warrants further studies in the future.

The present study is the first to assess the prognostic impact of AF on the mortality outcomes of a well-defined cohort of patients with confirmed acute PE using statewide population linkage analysis. It has previously been observed in smaller cohorts that AF could be an independent predictor of increased mortality in patients with PE [3,27]. Barra et al reported in a cohort of 270 patients admitted for acute PE that those with a history of AF (n = 57) had significantly higher mortality rates up to 6 months post PE compared to those without AF, independent of age, NT-proBNP, renal function and admission hemodynamics [27]. In our much large cohort of confirmed PE patients, when adjusted for differences in baseline characteristics including age, comorbidities and other parameters, we found only a trend towards poorer survival for the baseline AF group compared to the other groups. This may be partially explained by the relatively small proportion of deaths attributed to cardiovascular causes (35%), which may have reduced the overall impact of AF on all-cause mortality. On the other hand, cause-specific mortality analysis showed that both AF groups had proportionally significantly higher cardiovascular (especially acute myocardial infarction and heart failure) causes of death compared to the no AF group, indicating the adverse prognostic impact of AF is on cardiovascular outcomes in patients with acute PE.

The clinical relevance of the current study lies in the fact that the global burden of AF is increasing with population ageing [9]. The CARDIO-FIT observational study of 308 obese individuals with AF showed that patients with higher cardiorespiratory fitness gained from a tailored exercise program had significantly higher arrhythmia-free survival and lower AF burden and symptom severity after 4 years [28]. Early detection of and intervention for AF in patients with PE may likewise have a beneficial effect on morbidity.

The substantially high prevalence of baseline AF in patients with acute PE also raises concern about adequate anticoagulation in patients with AF. Only 9% of patients with baseline AF were anticoagulated with warfarin on admission in our study. In addition, patients presenting with PE despite being on warfarin therapy for baseline AF may indicate non-adherence or resistance to warfarin therapy. Our group previously reported a greater than 4-fold increased risk of death due to recurrent PE for patients who presented with acute PE despite taking warfarin on admission [29]. Whether the use of other anticoagulants such as the direct anti-thrombin or factor X inhibitors will prove to be more beneficial in this group of patients is not known.

The main limitation of our study comes from its retrospective and single-center design and our cohort did not include patients that died of PE before hospital presentation or had small PEs not requiring hospital admission. Despite the high prevalence and incidence rates of AF reported in the present study using a population-wide linkage, these rates nonetheless, likely represent underestimations in patients with PE given the paroxysmal nature of AF in some patients resulting in missing events. The reliance on ICD-10 coding to identify AF patients will be subject to potential coding errors (e.g. any history of arrhythmia possibly being coded as AF). In addition, we could not adjust for the impact of anticoagulation use during follow-up on mortality outcomes. Our classification of causes of death was based on the death certificate and follows guidelines from the World Health Organization. As a result, some causes of death could be misclassified in the absence of formal autopsy studies.

In summary, we report a very high prevalence of AF and a high incidence of subsequent AF in patients presenting with PE. Although all-cause mortality did not differ between patients with AF and those without AF after adjusting for differences in patients’ baseline characteristics, screening for AF may still be of importance post-PE due to its substantial impact on cardiovascular cause-specific deaths.

## Supporting Information

S1 Dataset (XLSX)

S1 Fig Adjusted survival curves. The unbroken line (Group 1) shows the survival curve of patients with no atrial fibrillation (AF) at baseline of index PE admission or during study follow-up period. The thick broken line represents patients with known AF at index PE admission (Group 2), while the dotted line represents patients who developed subsequent AF following index PE admission (Group 3). The survival curves are adjusted for age, gender, comorbidities based on Charlson Comorbidity Index, patient’s admission hemodynamic (heart rate, systolic blood pressure and oxygen saturation) and blood profiles (day-1 serum sodium, hemoglobin and estimated glomerular filtration rate). No significant differences in survival were observed across the three groups.
(TIFF)

S1 Table Univariable predictors for subsequent AF following acute PE presentation. (DOCX)

S2 Table Causes of death. (DOCX)
