# Supplementary figures and images for: The Prevalence and Incidence of Atrial Fibrillation in Patients with Acute Pulmonary Embolism
Source: PLoS One. 2016 Mar 1;11(3):e0150448. doi: 10.1371/journal.pone.0150448 (PMC4773188; doi:10.1371/journal.pone.0150448)

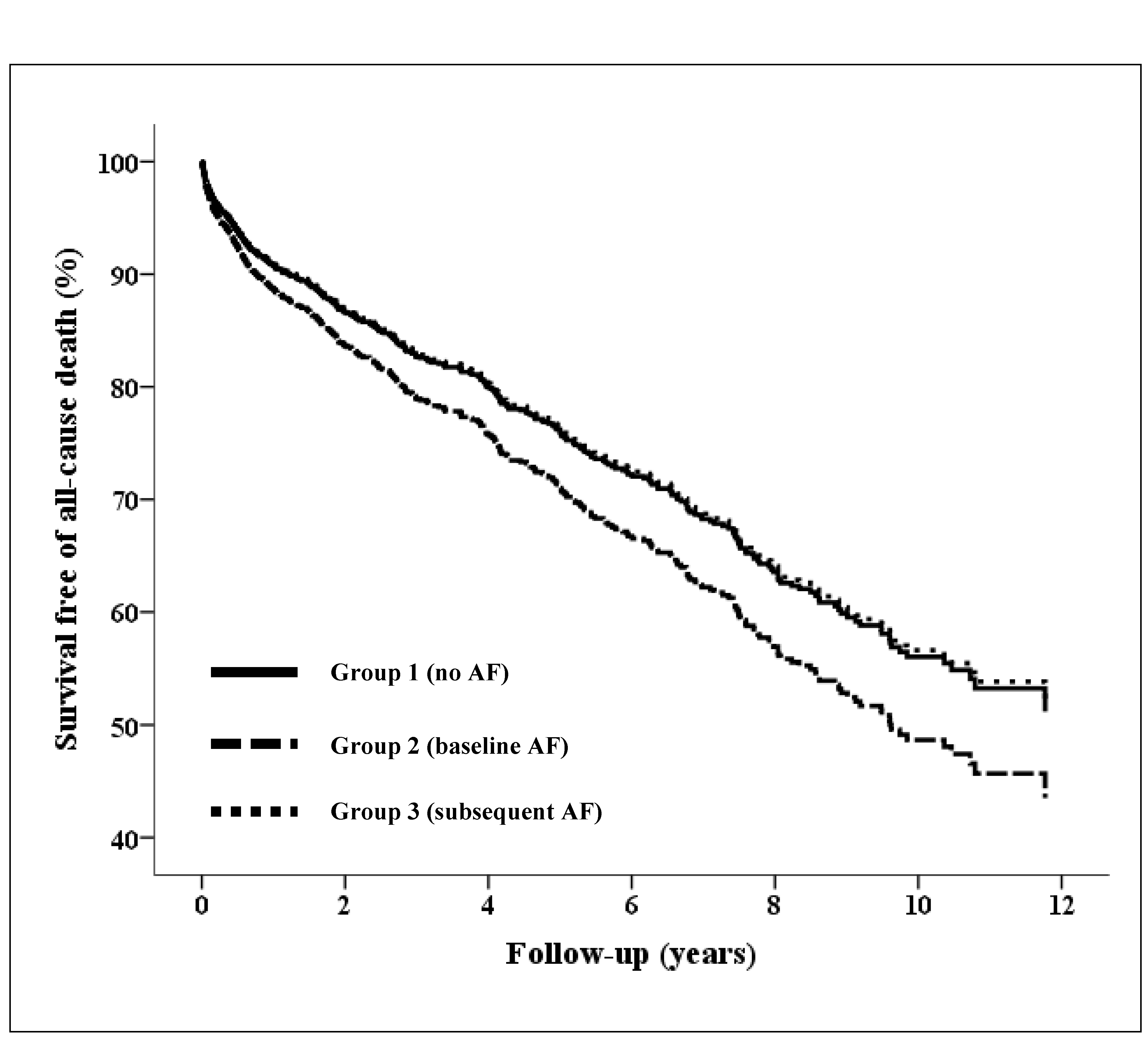

Supplement: S1 Fig — The unbroken line (Group 1) shows the survival curve of patients with no atrial fibrillation (AF) at baseline of index PE admission or during study follow-up period. The thick broken line represents patients with known AF at index PE admission (Group 2), while the dotted line represents patients who developed subsequent AF following index PE admission (Group 3). The survival curves are adjusted for age, gender, comorbidities based on Charlson Comorbidity Index, patient’s admission hemodynamic (heart rate, systolic blood pressure and oxygen saturation) and blood profiles (day-1 serum sodium, hemoglobin and estimated glomerular filtration rate). No significant differences in survival were observed across the three groups. (TIFF) [file pone.0150448.s002.tiff]
