# Supplementary material for: The Prevalence and Incidence of Atrial Fibrillation in Patients with Acute Pulmonary Embolism
Source: PLoS One. 2016 Mar 1;11(3):e0150448. doi: 10.1371/journal.pone.0150448 (PMC4773188; doi:10.1371/journal.pone.0150448)
Supplement: S1 Table — (DOCX) [file pone.0150448.s003.docx]

**S1 Table. Univariable predictors for subsequent AF following acute PE presentation.**

| **Parameters** | **Hazard ratio** | **95% confidence interval** | ***p* value** |
| --- | --- | --- | --- |
| Age, per-1-year increase | 1.06 | 1.05 – 1.08 | <0.001 |
| Males | 1.30 | 0.92 – 1.85 | 0.14 |
| Recorded DVT during admission | 0.99 | 0.65 – 1.52 | 0.97 |
| **Hemodynamic profile on admission** |  |  |  |
| Heart rate, per-1beat/minute increase | 1.00 | 0.99 – 1.01 | 0.92 |
| Systolic blood pressure, per-1mmHg increase | 1.00 | 0.99 – 1.01 | 0.62 |
| Oxygen saturation, per-1% increase | 0.99 | 0.95 – 1.02 | 0.46 |
| **Comorbidities, no. (%)** |  |  |  |
| Cardiovascular disease | 2.76 | 1.94 – 3.92 | <0.001 |
| Prior myocardial infarction | 2.28 | 1.53 – 3.41 | <0.001 |
| Prior CABG or PCI | 1.78 | 0.90 – 3.51 | 0.095 |
| Congestive cardiac failure | 3.53 | 2.16 – 5.76 | <0.001 |
| Valvular heart disease | 1.84 | 0.59 – 5.79 | 0.30 |
| Peripheral vascular disease | 1.54 | 0.91 – 2.61 | 0.11 |
| Stroke | 1.55 | 0.57 – 4.19 | 0.39 |
| Prosthetic valve | 2.50 | 0.35 – 17.9 | 0.36 |
| Cardiac risk factors |  |  |  |
| Hypertension | 2.07 | 1.44 – 2.97 | <0.001 |
| Hyperlipidemia | 1.69 | 1.06 – 2.67 | 0.03 |
| Diabetes | 2.09 | 1.37 – 3.19 | 0.001 |
| Current smoker | 0.88 | 0.50 – 1.56 | 0.66 |
| Ex-smoker | 1.35 | 0.88 – 2.07 | 0.17 |
| Noncardiovascular disease |  |  |  |
| Malignancy | 1.44 | 0.92 – 2.27 | 0.12 |
| Chronic pulmonary disease | 1.64 | 0.98 – 2.73 | 0.06 |
| Obstructive sleep apnea | 2.32 | 0.86 – 6.28 | 0.098 |
| Dementia | 0 | 0 | - |
| Parkinson’s disease | 1.41 | 0.35 – 5.74 | 0.63 |
| Chronic renal disease | 0.93 | 0.34 – 2.53 | 0.89 |
| Charlson Comorbidity Index, per-1-score increase | 1.15 | 1.06 – 1.25 | 0.001 |
| **Blood profile on admission** |  |  |  |
| Day-1 sodium, per-1mmol/L increase | 0.91 | 0.87 – 0.96 | <0.001 |
| Day-1 eGFR, per-1ml/min/1.74m^2^ increase | 0.99 | 0.98 – 0.99 | <0.001 |
| Day-1 hemoglobin, per-1g/L increase | 0.99 | 0.98 – 1.00 | 0.02 |
| **Medications use on admission, no. (%)** |  |  |  |
| Beta-blocker | 0.87 | 0.53 – 1.43 | 0.59 |
| AF, atrial fibrillation; CT, computed tomography; CABG, coronary artery bypass grafting; PCI, percutaneous coronary intervention; DVT, deep vein thrombosis; PE, pulmonary embolism; Estimated glomerular filtration rate (eGFR) = 186 x ([S_CR_/88.4]^-1.154^) x (age)^-0.203^ x (0.742 if female), where estimated GFR = estimated glomerular filtration rate (ml/min/1.73m2), S_CR_ = serum creatinine concentration (µmol/L), and age is expressed in years. | | | |
