# Supplementary material for: The Prevalence and Incidence of Atrial Fibrillation in Patients with Acute Pulmonary Embolism
Source: PLoS One. 2016 Mar 1;11(3):e0150448. doi: 10.1371/journal.pone.0150448 (PMC4773188; doi:10.1371/journal.pone.0150448)
Supplement: S2 Table — (DOCX) [file pone.0150448.s004.docx]

**S2 Table. Causes of death.**

| **Causes of death, no. (%)** | **Whole cohort**  **N=478** | **Group 1**  **No AF**  **N=283** | **Group 2**  **Baseline AF**  **N=119** | **Group 3**  **Subsequent AF**  **N=76** |
| --- | --- | --- | --- | --- |
| **Cardiovascular causes** | 167 (35) | 73 (26) | 59 (50)* | 35 (46)* |
| Pulmonary embolism | 47 (10) | 26 (9) | 16 (13) | 5 (7) |
| Acute myocardial infarction | 29 (6) | 11 (4) | 11 (9)* | 7 (9) |
| Heart failure | 39 (8) | 13 (5) | 13 (11)* | 13 (17)* |
| Stroke | 23 (5) | 10 (4) | 7 (6) | 6 (8) |
| Cardiac arrest | 9 (2) | 5 (2) | 4 (3) | 0 |
| Cardiac-related‡ | 20 (4) | 8 (3) | 8 (7) | 4 (5) |
| **Noncardiovascular causes** | 311 (65) | 210 (74) | 60 (50)* | 41 (54)* |
| Sepsis | 105 (22) | 61 (22) | 21 (18) | 23 (30)† |
| Malignancy | 137 (29) | 109 (39) | 17 (14)* | 11 (15)* |
| Others | 53 (11) | 30 (11) | 17 (14) | 6 (8) |
| Undefined | 16 (3) | 10 (4) | 5 (4) | 1 (1) |
| - *p*<0.05 compared to Group 1 (control group) - *p*<0.05 compared to Group 2 (baseline AF group) - Cardiac-related cause of death is coded when more than one cardiac cause of death is recorded on the death certificate. | | | | |
